# Learning and visualizing chronic latent representations using electronic health records

**DOI:** 10.1186/s13040-022-00303-z

**Published:** 2022-09-05

**Authors:** David Chushig-Muzo, Cristina Soguero-Ruiz, Pablo de Miguel Bohoyo, Inmaculada Mora-Jiménez

**Affiliations:** 1 Department of Signal Theory and Communications, Telematics and Computing Systems, Rey Juan Carlos University, Madrid, Spain; 2 University Hospital of Fuenlabrada, Madrid, Spain

**Keywords:** Denoising Autoencoder, Chronic diseases, Diabetes, Hypertension, Clustering, Patient representation, Synthetic patient, Health status trajectory

## Abstract

**Background:**

Nowadays, patients with chronic diseases such as diabetes and hypertension have reached alarming numbers worldwide. These diseases increase the risk of developing acute complications and involve a substantial economic burden and demand for health resources. The widespread adoption of Electronic Health Records (EHRs) is opening great opportunities for supporting decision-making. Nevertheless, data extracted from EHRs are complex (heterogeneous, high-dimensional and usually noisy), hampering the knowledge extraction with conventional approaches.

**Methods:**

We propose the use of the Denoising Autoencoder (DAE), a Machine Learning (ML) technique allowing to transform high-dimensional data into latent representations (LRs), thus addressing the main challenges with clinical data. We explore in this work how the combination of LRs with a visualization method can be used to map the patient data in a two-dimensional space, gaining knowledge about the distribution of patients with different chronic conditions. Furthermore, this representation can be also used to characterize the patient’s health status evolution, which is of paramount importance in the clinical setting.

**Results:**

To obtain clinical LRs, we considered real-world data extracted from EHRs linked to the University Hospital of Fuenlabrada in Spain. Experimental results showed the great potential of DAEs to identify patients with clinical patterns linked to hypertension, diabetes and multimorbidity. The procedure allowed us to find patients with the same main chronic disease but different clinical characteristics. Thus, we identified two kinds of diabetic patients with differences in their drug therapy (insulin and non-insulin dependant), and also a group of women affected by hypertension and gestational diabetes. We also present a proof of concept for mapping the health status evolution of synthetic patients when considering the most significant diagnoses and drugs associated with chronic patients.

**Conclusion:**

Our results highlighted the value of ML techniques to extract clinical knowledge, supporting the identification of patients with certain chronic conditions. Furthermore, the patient’s health status progression on the two-dimensional space might be used as a tool for clinicians aiming to characterize health conditions and identify their more relevant clinical codes.

**Supplementary Information:**

The online version contains supplementary material available at (10.1186/s13040-022-00303-z).

## Introduction

Nowadays, the global aging phenomenon has become an important health challenge. It is a common ground that elderly people are more likely to develop chronic diseases [1]. The increase in patients suffering from chronic diseases is one of the major challenges faced by National Health Systems, especially in low-income and middle-income countries [2]. Current societies are characterized by following unhealthy habits. Overweight, obesity, and physical inactivity are main factors for developing chronic conditions. Diabetes and hypertension are relevant chronic conditions that augment the risk of complications, becoming major risk factors for developing acute complications that lead to disability and mortality [3]. A high number of chronic patients suffer from multimorbidity, defined as the co-occurrence of (multiple)-chronic diseases [4]. Multimorbidity becomes progressively more common with age, and patients tend to have psychological distress, complex drug treatments, and longer hospital stays, impairing their quality of life [5, 6]. The World Health Organization has emphasized the importance of organizing healthcare delivery systems to improve health outcomes and has stressed the importance of building integrated healthcare systems that can address chronic disease management. Over the last years, key policy makers and health stakeholders have looked for strategies aiming to efficiently allocate health resources for the care of chronic patients. Among them, early identification could support the implementation of appropriate treatment and better care management.

With the widespread adoption of Electronic Health Records (EHRs), large volumes of data in a variety of formats and sources have been collected [7]. The use of EHRs is bringing great opportunities for clinical research, fostering the development of data-driven approaches, especially those based on Machine Learning (ML) techniques. ML is a branch of Artificial Intelligence seeking to provide knowledge from experience (data) [8]. EHRs, in conjunction with ML, have been successfully used in different clinical areas [9–11], shifting from expert-driven approaches to data-driven models. Within ML, unsupervised learning methods aim to reveal underlying patterns from data and group similar samples. In the clinical setting, these methods can be used to identify and group patients with similar health statuses, specially groups associated with chronic conditions.

Despite the benefits of applying ML in the clinical setting, data extracted from EHRs are heterogeneous and characterized by a high number of features. Also, the limited number of observations in the clinical real-world scenario is a challenge when dealing with high-dimensional data, what is referred to as the curse of dimensionality problem [12]. This hampers data visualization, knowledge discovery and the effective application of traditional ML approaches in healthcare [13, 14]. To tackle this issue, different linear and non-linear methods transforming the original high-dimensional data into low dimensional representations have been proposed [15, 16]. Among these models, the Autoencoder (AE) [17] seeks to address the high-dimensionality issue by building lower-dimensional representations, named latent representations (LRs). The LRs capture relevant relationships from data and support the application of subsequent ML tasks such as data visualization and clustering.

In recent years, several regularization strategies (imposing some restrictions on the AE) have been proposed for increasing the robustness and generalization of the LRs built with AE-based models. This has yielded new types of AEs [18–22]. Among them, the DAE [18] is a regularized AE that seeks to build more robust LRs with the inclusion of noise through a stochastic corruption process applied at the network input. The implicit regularization process performed by DAEs when corrupting input data with noise allows it the capability to provide robust representations [19]. In the clinical setting, several studies have successfully applied AE-based models in different applications [23–30]. For instance, in [25], patient representations were built from raw EHRs through a three-layer stacked AE for disease risk prediction using random forests as classifiers. Towards that end, EHR-based data of approximately 700,000 patients diagnosed with schizophrenia, diabetes, and different types of cancer were used. Stacked DAEs were used for identifying patterns of physiology in clinical time series [23]. In [24], a stacked AE was used to model longitudinal sequences of serum uric acid measurements to distinguish between the uric-acid signatures of gout and acute leukemia. A stacked DAE with sparsity constraint was used for classifying ECG signals in [27]. A combination of Stacked AEs and Convolutional Neural Networks was applied to classify EEG signals in [26]. In [31], a novel AE named long short-term memory convolutional autoencoder LSTMCAE was proposed to learn feature representations from sensor signals for improving health condition monitoring. The authors in [28] used single-layer DAE for encoding patient records composed of synthetic clinical descriptors and applied t-SNE for phenotype stratification.

The contribution of this paper is twofold. First, we analyze the potential of using the DAEs for building LRs to characterize chronic patients using binary data associated with diagnoses and drug codes. These LRs serve as a base for finding groups of patients with similar clinical conditions, while visualizing high-dimensional data into a lower-dimensional space, thus supporting the clinical interpretation. Second, we present a proof-of-concept to determine the relevant codes for a clinical condition and to visually characterize the health status evolution of synthetic patients on the low-dimensional space. In this work, we used data of healthy and chronic patients (including hypertensive, diabetics, and multimorbidity patients) corresponding to real-world EHRs of the University Hospital of Fuenlabrada (UHF) of Madrid, Spain.

The remainder of this paper is organized as follows: Data description and the preprocessing stage are detailed in the next section. Then, we describe the theoretical fundamentals of DAEs as well as the clustering methods used. Next, we present the results of this work, showing the clinical characterization of the groups of patients found. Also, a proof-of-concept aiming to visually characterize the health status progression of chronic patients is presented. Finally, the discussion and conclusions are provided in the last part of the manuscript.

## Materials and methods

### Data description and preprocessing

In this subsection, we describe the dataset used in this study as well as the pre-processing carried out. We considered clinical data of healthy and chronic patients assigned to the UHF. The UHF is a public hospital providing medical service to six health centers and encompasses about 225,000 inhabitants. Patient data included demographics (age and sex), diagnoses from primary and specialized care and pharmaceutical drug dispensation. The diagnoses were coded according to the International Classification of Diseases-Ninth Revision-Clinical Modification (ICD9-CM) [32], while pharmaceutical drug codes followed the Anatomical Therapeutic Chemical (ATC) Classification System [33].

The ICD9-CM and ATC codes have been extensively used in a variety of studies at the international level [34, 35]. Both kinds of codes are hierarchically structured and composed of a different number of alphanumeric characters (ANCs). The ICD9-CM codes have from three to five ANCs with a decimal point between the third and fourth character. Regarding the ATC codes, they are identified by seven ANCs, structured in five levels: (1) anatomical (first element), (2) therapeutic (second and third element), (3) pharmacological (fourth element), (4) chemical (fifth element), and (5) chemical substance (sixth and seventh element). Following a similar approach to [36], we reduced the detail of the aforementioned codes by discarding the ANC after the decimal point for ICD9-CM and discarding the fifth level for ATC codes. Every patient is represented by one vector composed of 2,263 clinical features (1,517 ICD9-CM codes, and 746 ATC codes) that take into account only the presence/absence of codes for one year. In our case, we identify the presence/absence of a specific diagnosis or drug code by binary values ‘1’ and ‘0’, respectively. The features linked to diagnoses take the value ‘1’ when the patient has been diagnosed with a specific disease/pathology, and ‘0’ otherwise. Regarding drugs, features coded as ‘1’ indicate that the particular drug has been dispensed to the patients (one or more times during a year), otherwise the feature is coded by ‘0’. The clinical codes were used for training the DAEs, while the demographics age and sex were just used for characterizing the clusters found.

Nowadays, with the increasing prevalence of chronic diseases, many countries have carried out a variety of strategies aiming to efficiently allocate health resources. Among these approaches, the system named Clinical Risk Groups (CRGs) [37] is a solution clinically validated and mainly focused on the identification of chronic patients [38–41]. CRGs are a population classification system that assigns each individual to only one of a set of pre-established groups (more than 1,0000) by taking into account the demographic and clinical features. Each group is described by a five-digit number. The first digit refers to the core health status group, the next three digits identify the base CRG representing a specific condition, and the last digit denotes the severity-of-illness level. In order to have a reasonable number of patients per group and following the same approach that in previous works [36, 42], we discarded the fifth digit (severity level) and considered the collection of patients provided by the base CRG. Then, we used CRGs (version 1.8) to identify healthy and chronic patients belonging to the UHF. In particular, we work with the next CRGs: CRG-1000 (encompassing 46,835 healthy patients), CRG-5192 (12,447 hypertensive patients), CRG-5424 (2,166 diabetic patients), and CRG-6144 (composed of 3,179 patients suffering from multimorbidity, co-occurring diabetes and hypertension). Since the number of patients associated with each CRG is highly imbalanced and ML algorithms are affected by the class imbalance learning problem, we followed a random under-sampling strategy as in [36]. Thus, the number of patients considered per CRG is limited by the size of CRG-5424 (2,166 patients).

**Fig. 1:**
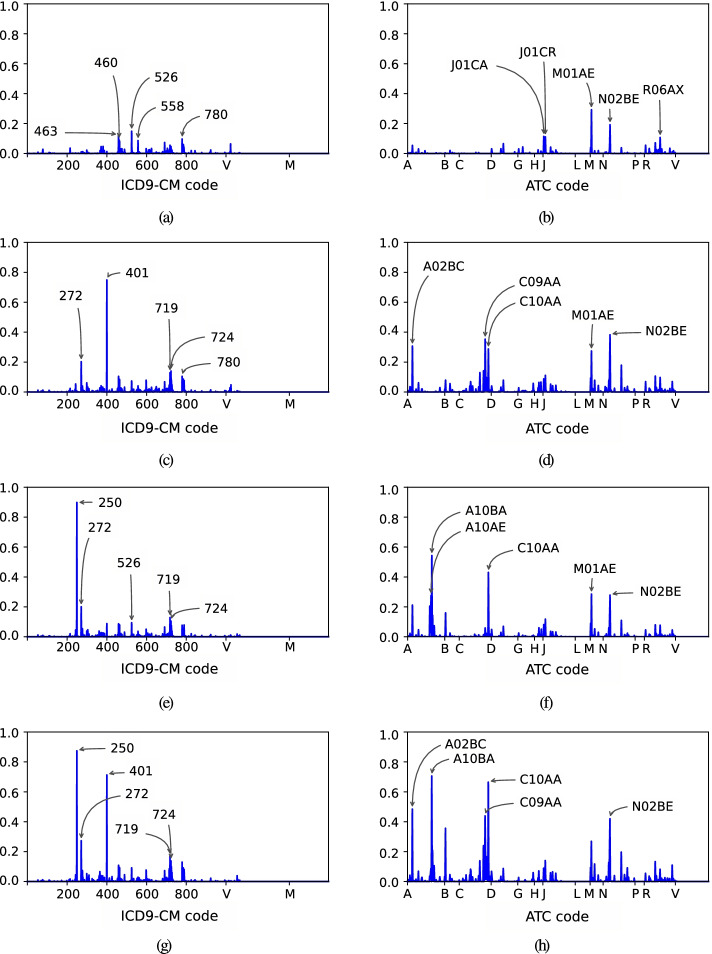
Profiles associated with the CRGs when considering the dataset provided by the UHF. Diagnosis (left panels) and drug profiles (right panels) associated with (**a**-**b**) CRG-1000; (**c**-**d**) CRG-5192; (**e**-**f**) CRG-5424; (**g**-**h**) CRG-6144

To provide a visual representation of the code distribution per health status associated with the CRGs, we use the profile [36]. The profile is a bar graph where the x-axis shows the clinical codes (ICD9-CM or ATC codes) and the y-axis presents the corresponding presence rate (ranging between [0,1]). The profiles associated with the CRGs considered in this work are shown in Fig. 1. With *n* patients, the corresponding profile is built from the concatenation of 2,263 elements (codes of diagnoses and drugs). For the d-th element in the profile (*e.g.,* assume it is associated with the hypertension diagnosis), its value is computed as the proportion of *n* patients presenting the particular diagnosis code of hypertension (ICD9-CM ‘401’). Thus, profiles with elements close to 1 indicate that almost all patients have the same clinical code (either diagnosis or drug). Thus, the profile provides an overall vision of the most representative diagnoses and drugs linked to one group of patients. Note that the five codes with the highest presence rate values were pointed out in Fig. 1. Taking as an example the diagnosis profile of CRG-5424 (see Fig. 1 (e)), the code with the highest presence rate is ICD9-CM ‘250’ (Diabetes Mellitus) with a value over 0.8, indicating that about 80% of patients belonging to this CRG present this code in the EHR. The drug profile of CRG-5424 (see Fig. 1 (f)) showed that the most dispensed drugs correspond to ATC ‘A10BA’ (biguanides) and ‘C10AA’ (HMG CoA reductase inhibitors). The first drug is frequently used in the treatment of diabetes, whereas the second one is utilized for reducing cholesterol levels.

### Methods

In this subsection, we describe the AE-based models and clustering methods used in this paper.

#### Learning clinical latent representations based on autoencoders

Let $\mathcal {X}=\{\mathbf {x}^{(i)}\}_{i=1}^{n}$ be a dataset consisting of *n* samples, with the *i*-th sample (patient in this paper) represented by a vector of *D* features, $\mathbf {x}^{(i)}=\left [x_{1}^{(i)}, \dots, x_{D}^{(i)}\right ] \in \mathbb {R}^{D}$. We use AEs to transform **x** into a LR named $\mathbf {h}~=~[h_{1},\dots,h_{d}] \in \mathbb {R}^{d}$, composed of *d* latent dimensions. Note that the dimension of **x** is higher than that of **h**, fulfilling *D*>*d*. Next, we introduce the fundamentals of the AE-based models, as well as those of the clustering methods used in this work.

**Fig. 2:**
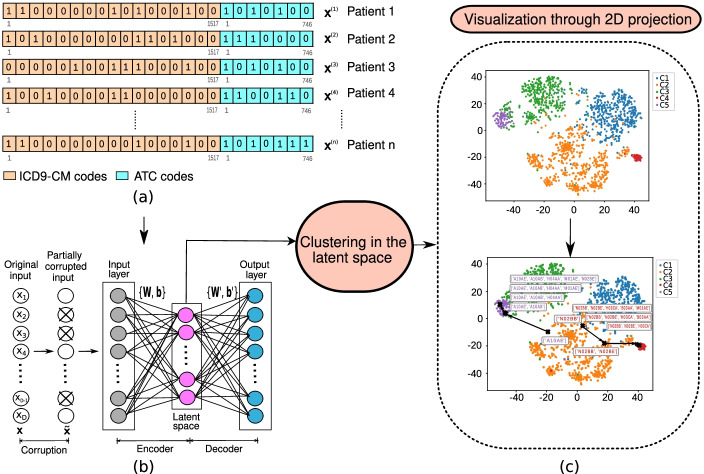
Schematic representation of the proposed workflow. (**a**) Representation of *n* patient vectors with their corresponding clinical features (diagnosis and drug codes, with binary values); (**b**) DAE architecture, and (**c**) clustering on the LRs and subsequent visualization through two-dimensional projections, both of the clusters and of the patient progression

An AE is a fully connected artificial neural network performing an encoding-decoding process through non-linear transformations [17]. The simplest AE consists of three layers (input, hidden and output layer) composing the encoder and decoder (see schematic in Fig. 2 (a)). The output of the encoder provides a lower-dimensional representation by transforming an input vector **x** into a dimensionality-reduced representation **h** guided by the mapping:

$$h(\mathbf{x}) = f(\mathbf{W} \mathbf{x} + \mathbf{b})$$

where $\mathbf {W} \in \mathbb {R}^{d \times D}$ is a weight matrix and $\mathbf {b} \in \mathbb {R}^{d}$ is a bias vector associated with the encoder. Since the number of neurons of the hidden layer is lower than the number of neurons of the input layer, the network is forced to learn a compressed representation of the input. Thus, the encoding process reduces high-dimensional data to low-dimensional data (compressed representation named LR), creating a new feature space called latent space. Next, the decoder seeks to reconstruct **x** by transforming **h** to the vector $\hat {\mathbf {x}} \in \mathbb {R}^{D}$ guided by:

$$\hat{\mathbf{x}} = g(\mathbf{W}^{\prime} \mathbf{h} + \mathbf{b}^{\prime})$$

where $\mathbf {W}^{\prime } \in \mathbb {R}^{D \times d}$ is a weight matrix and $\mathbf {b}^{\prime } \in \mathbb {R}^{D}$ a bias vector associated with the decoder. The functions *f*(·) and *g*(·) are non-linear functions (commonly a sigmoid or hyperbolic function) parameterized by *θ* = {**W**,**b**} and *θ*^′^ = {**W**^′^,**b**^′^}. The AE is trained by adjusting the parameters *θ*,*θ*^′^ to minimize the difference between **x** and $\hat {\mathbf {x}}$ through a cost function. In this work, the binary cross-entropy function has been considered due to the binary nature of the features in **x**.

In order to build more robust LRs, different regularization techniques can be applied. Specifically, we construct an AE with a three-layer neural network with stochastic noise at the network input [18]. Thus, we work with a noisy version of the inputs by corrupting the input samples in a controlled manner (see schematic in panel (b) of Fig. 2). The purpose of using DAE is twofold: *(i)* to learn more robust latent representations; and *(ii)* to reduce the risk of overfitting, that can be problematic with regular AE. Previous works have used different corruption strategies, such as Zero Masking Noise (ZMN) and Salt-and-Pepper Noise (SPN) [18, 19]. Both strategies corrupt a fraction of the features (level of noise) of an input sample, forcing the value to the minimum in ZMN and setting the value to the minimum/maximum value (according to a fair coin flip) in SPN [18]. For binary features in **x**, *i.e.,* *x*_*i*_∈{0,1}∀*i*=1,…,*D*, the maximum and minimum value corresponds with binary values ‘1’ or ‘0’, respectively. As stated, we work with binary data, and both ZMN and SPN are considered and compared in this paper.

#### Clustering methods

Clustering aims to find partitions (best known as clusters) of a dataset such that each cluster is composed of similar samples according to a similarity measure [12], being the Euclidean distance the most common one [43]. Formally, given a dataset $\mathcal {X}$ with *n* patients, a clustering method results into *n*_*k*_ disjoint groups denoted by $\phantom {\dot {i}\!}\mathcal {C}=\{C_{i}\}_{i=1}^{n_{k}}$, where *C*_*i*_ denotes the *i*-th cluster containing *n*_*i*_ patients. In this paper, the LRs are used for performing clustering methods. Clustering methods have many practical applications in different domains, including image processing and pattern recognition, among others [44]. When considering ML approaches in the clinical setting, each patient may be represented by a sample with different features, including demographics (age, sex) and clinical codes (diagnosis codes, drug codes), among others. The patients with the same health condition present common clinical characteristics and patterns, and their early identification could support the implementation of appropriate treatment and better care management.

In the literature, a plethora of clustering methods has been proposed [44], being the partitioning and hierarchical approaches the most popular ones [45]. Partitioning methods are frequently selected due to ease of implementation and lower computational cost [45]. However, these methods are highly sensitive to outliers, cannot find non-convex clusters, and are highly dependent on the initial conditions [45]. Hierarchical clustering methods overcome these difficulties at the expense of higher computational costs. Both clustering approaches have been applied in a wide range of fields including healthcare, data mining, and natural language processing, among others [45]. The most used partitioning method is *k*-means [46], where each cluster is represented by a centroid or representative vector. In the *k*-means algorithm, the number of *k* clusters is established a priori, and the centroid’s location is found by minimizing a cost function that takes into account the average intra-cluster distance between samples and associated centroids [45]. By contrast, algorithms used in hierarchical clustering approaches seek to build a hierarchy of clusters according to both agglomerative and divisive strategies. The Agglomerative Hierarchical Clustering (AHC), with the commonly used Ward linkage, has been considered in this study. It starts by considering each sample as a single cluster. At each iteration of the algorithm, the two clusters with the lowest Ward linkage are merged into a new cluster [47]. Several studies have used the AHC [48, 49] and *k*-means [50, 51] in clinical applications, providing good clustering outcomes. We evaluate and compare the clustering results of both *k*-means and the AHC method in this paper.

It is broadly known that the major challenge in clustering is the choice of an appropriate number of clusters. Many methods have been proposed for addressing this issue, being the cluster validity indices (CVIs) the most extended measures [52]. The CVIs aim to measure how closely are the samples in the cluster (compactness) and how separated a cluster is from other clusters (separability or inter-variance) [52]. Among the CVIs proposed in the literature [52–54], in this study, we considered the silhouette coefficient, the Davies-Bouldin index and the Bayesian inference criterion (BIC) to select the number of clusters because they quantify the intra-cluster (compactness) and inter-cluster (separability) distances. This means that these indices estimate how compact are samples within their corresponding clusters, and how separated are the clusters between them. Regarding the silhouette coefficient, their values range between [−1,1], with 1 indicating the best clustering performance, values close to 0 denote overlapping clusters, and -1 means the worst clustering performance. For the Davies-Bouldin index, smaller values indicate a better clustering result. A small value of BIC mean more compact clusters, indicating better clustering. The optimal number of clusters is chosen as the value such that the BIC is minimized.

## Results

### Experimental setup

We considered two case studies. The first one takes into account healthy (CRG-1000) and chronic patients with just one major chronic condition: hypertension (CRG-5192) or diabetes (CRG-5424). The second case-study also includes multimorbid patients who suffer from hypertension and diabetes (CRG-6144). As previously stated, CRGs present remarkable differences in the number of patients, emerging the class imbalance problem. To deal with this issue, several approaches have been presented in the literature [55]. For simplicity, an under-sampling approach was followed in this work, which randomly selects a subset of patients in each CRG according to the minority class. In our case, the CRG-5424 corresponds to the minority class with 2,166 patients. This yields a total of 6,498 patients in the first case-study and 8,664 in the second one. These patients are characterized by binary features coding the presence or absence of diagnosis and drug codes which were used as input for DAEs. The resulting datasets were split into training and test subsets. For each case-study, we randomly consider 75% of patients for the training subset and the remaining for testing. Note that each patient is only considered in one subset (training or testing). The test subset was composed of 1,625 and 2,166 patients for the first and second case study, respectively. The training subset was used to design the DAEs and select the number of clusters with the cluster validity indices, while the test subset was considered for the clinical characterization of the found clusters. As stated, each patient is represented by one vector composed of 2,263 clinical features (1,517 ICD9-CM codes, and 746 ATC codes) that take into account only the presence/absence of codes for one year.

DAEs were trained with a mini-batch gradient descent with adaptive learning rate and early stopping [55]. Rectified linear activation functions were considered for all neurons except those in the output layer, where a sigmoid was used. Other activation functions such as RELU and ELU [56] also considered (but its use was empirically discarded). Following a similar approach to [18], we evaluated different types of noise (ZMN and SPN), several levels of noise {10,50,100}, a different number of neurons in the hidden layer {5,10,20,30} and the addition of a new hidden layer (between input and intermediate layer) with different numbers of neurons. Empirical results showed that SPN with *d*=20 and a corruption level of 10, and just the use of one hidden layer provided an appropriate architecture to find groups of patients with similar characteristics, and also to visualize patterns associated with different chronic diseases. Additionally, a comparative analysis between the DAE and linear dimensionality reduction methods (including principal component analysis and factor analysis) was conducted aiming to compare the performance for reducing dimensionality and visualizing high-dimensional data. These results were included in the additional information file 1.

### Estimation of the number of clusters

**Fig. 3:**
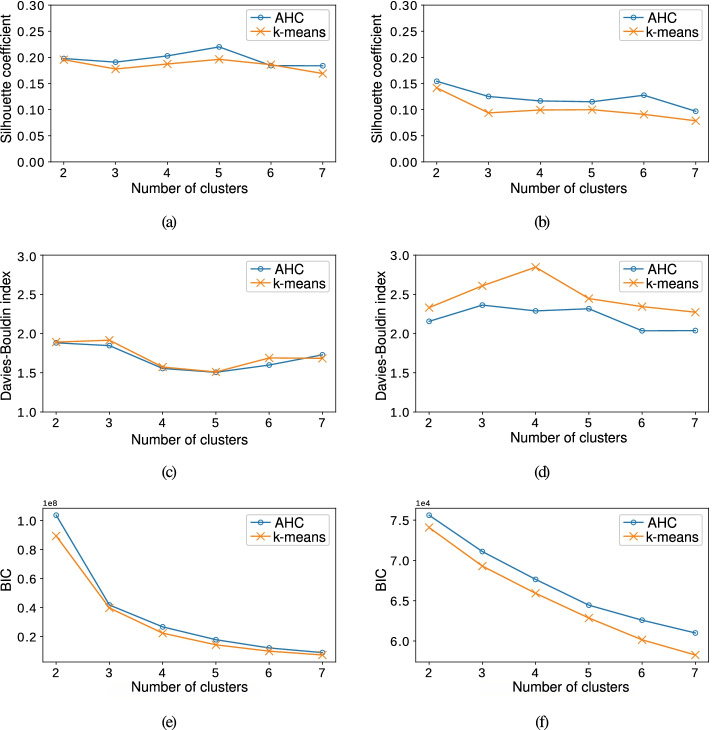
Cluster validity indices when considering both case studies (left panels) CRGs-1000-5192-5424 and (right panels) CRGs-1000-5192-5424-6144. (**a**-**b**) The silhouette coefficients; (**c**-**d**) the Davies-Bouldin indices; and the (**e**-**f**) BIC values

In this work, the LRs are proposed to discover clusters of patients with similar clinical characteristics by using both *k*-means and AHC with the Ward linkage. To estimate the number of clusters, the silhouette coefficient (see Fig. 3 (a-b)), the Davies-Bouldin index (see Fig. 3 (c-d)) and the BIC (see Fig. 3 (e-f)) were considered as CVIs. The CVIs for the first case-study with CRGs-1000-5192-5424 are shown in the lefts panels, whereas those for the second case-study considering CRGs-1000-5192-5424-6144 are depicted in the right panels.

According to Fig. 3, the AHC usually provided better performance than *k*-means, reaching the lowest values in the Davies-Bouldin index and the highest values in the silhouette coefficient and BIC values. Regarding the first case-study with CRGs-1000-5192-5424, both the Davies-Bouldin and the silhouette coefficient indices (see Fig. 3 (a) and (b), respectively) revealed that the most appropriate number of clusters was five. In the case of BIC, we considered the elbow point technique [57] that consists in plotting a metric (in our case, BIC) depending on the number of clusters, and finding the inflection point down. For the first case study (see Fig. 3, left panels), the elbow point was identified in five clusters. The clinical complexity of the second case-study, including patients suffering from multimorbidity, also exhibits greater complexity to identify the most appropriate number of clusters when considering CVIs. Thus, though the minimum value of the Davies-Bouldin index in Fig. 3 (d) indicated six as a suitable number of clusters, the silhouette coefficient in Fig. 3 (b) pointed out that two clusters should be the most appropriate. By considering the BIC (see Fig. 3 (f)), it was not straightforward to identify an elbow point since BIC values monotonically decreased with the number of clusters, becoming a smooth curve with no clear minimum. Taking into account that four CRGs are considered in the second case-study, the selection of two clusters may not be the best option to characterize the clinical conditions of these patients. By choosing the next highest value for the silhouette coefficient (which corresponds with six clusters) the number of clusters selected by CVIs is the same. Henceforth, we will consider the AHC method with five clusters for the first case-study, and with six clusters for the second case-study.

### Clinical characterization of clusters associated with healthy and chronic patients

**Fig. 4:**
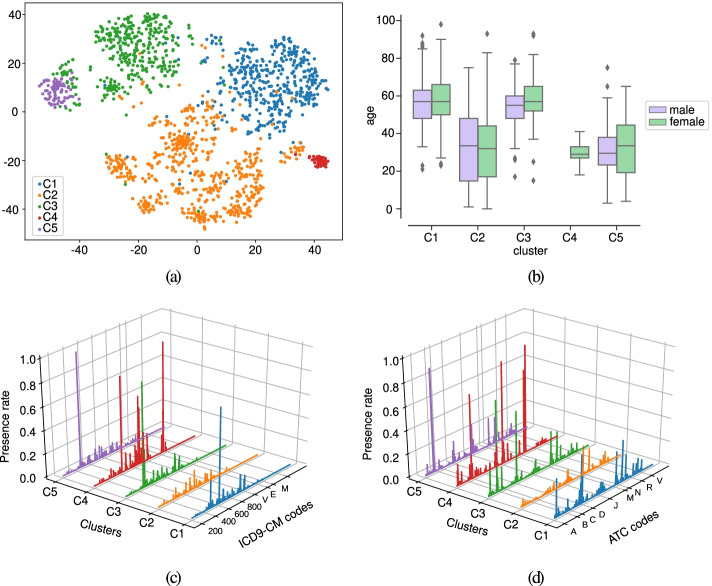
Analysis of clusters considering CRGs-1000-5192-5424 and the test subset. (**a**) Projection of patients by combining DAE, AHC, and t-SNE; (**b**) boxplot of the age for each cluster; profiles considering (**c**) diagnosis codes and (**d**) drug codes. Note that each color corresponding to a specific cluster

**Fig. 5:**
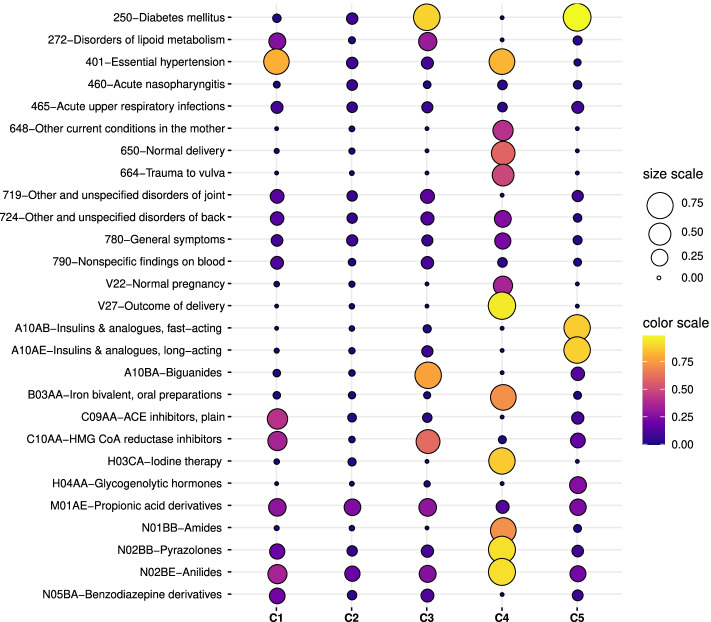
Description of the most frequent clinical codes for clusters found when considering CRGs-1000-5192-5424 and using the test subset. Each code is represented by a dot whose area and color is proportional to the presence rate

The characterization of the clusters for the first case-study is provided in this subsection. Following a similar approach to [24, 28], we used the LRs as input to the t-Stochastic Neighbor Embedding (t-SNE) [58] method aiming to visualize clusters of patients in two dimensions. This projection is depicted in Fig. 4 (a). Five identifiers are considered to distinguish the clusters: C1 (blue), C2 (orange), C3 (green), C4 (red), C5 (purple). We show in Fig. 4 (b) the boxplots of the age variable (not considered for training DAEs) per cluster. The drug and diagnosis profiles are displayed in Fig. 4 (c) and (d). The description of the clinical codes with the highest presence rate values are presented in the balloon plot of Fig. 5.

Regarding cluster C1, the diagnosis profile showed ICD9-CM codes ‘401’ and ICD9-CM ‘272’ as the two most frequent ones. The first is the main code used for coding hypertension, and the second one is associated with lipid disorders. By analyzing the drug profile, patients of cluster C1 were characterized by the consumption of ATC ‘C09AA’ (angiotensin-converting enzyme (ACE) inhibitors), ‘C10AA’ (HMG-CoA reductase inhibitors known as statins), ‘N02BE’, ‘M01AE’. Among them, ‘C09AA’ is the most frequent drug prescribed for hypertension, which aims to reduce both blood pressure and the risk of cardiovascular events (such as stroke, myocardial infarction, and heart failure) [59]. The ATC code ‘C10AA’ is a drug extensively used to prevent cardiovascular diseases by lowering serum cholesterol [60]. By analyzing the profiles in Fig. 4 (c-d), those associated with C1 are the most similar to the profiles of CRG-5192 (see Fig. 1 (c-d)). Table 1 also showed that cluster C1 was mainly composed of patients assigned to CRG-5192.

**Table 1:** Cluster ID, number and percentage of patients associated with CRG-1000, CRG-5192 and CRG-5424 for each cluster when considering test subset

	Cluster ID	# patients	CRG-1000	CRG-5192	CRG-5424
	C1	430	2	94	4
	C2	726	74	16	10
	C3	310	0	1	99
	C4	39	15	80	5
	C5	120	0	0	100

For cluster C2, the exploration of its diagnosis profile (see Fig. 4 (c)) did not show great differences in diagnosis codes with CRG-1000 (see Fig. 1 (a)). For drugs (see the profile in Fig. 4 (d)), we observed that the most common ATC codes were ‘N02BE’ (Anilides) and ‘M01AE’ (Propionic acid derivatives), which are analgesics. The analysis of cluster C2 showed that the majority of their associated patients were healthy. As it is denoted in Table 1, 10% patients are associated with CRG-5424, 16% with CRG-5192, and 74% with CRG-1000. By analyzing patients in C2 categorized as diabetics and hypertensives, we observed that they only had assigned ICD9-CM ‘250’ and ‘401’, but the medication found in the corresponding drug profile were analgesics (‘N02BE’ and ‘M01AE’), with a lack of anti-hypertensive or antihyperglycemic drugs. Thus, cluster C2 is characterized by a healthy population.

The diagnosis profiles of both C3 and C5 showed the prevalence of ICD9-CM codes ‘250’ (Diabetes Mellitus) (see Fig. 4 (c)). The drug profile of cluster C3 mainly showed the prevalence of ATC ‘A10BA’ (biguanides) and ATC ‘C10AA’. Biguanides are antihyperglycemic drugs used to improve glucose tolerance, being the most recommended treatment for type-II diabetes [61]. By contrast, patients of C5 showed high consumption of different types of insulin: ‘A10AB’ (fast-acting), and ‘A10AE’ (long-acting). The visual analysis of Fig. 4 (a) is particularly relevant because the scatter plot corroborates that diabetics were split into two clusters: C3 (green points) and C5 (purple points). Note that the profiles of C3 are quite similar to those of CRG-5424 (see Fig. 1 (e-f)). By analyzing Table 1 in detail, note that 99% patients of cluster C3 and 100% of cluster C5 come from CRG-5424. With this information, we can characterize C3 as diabetics who take mainly biguanides and cluster C5 as diabetics insulin-dependant.

A small group of patients identifying cluster C4 (red points) is depicted in the scatter plot of Fig. 4 (a). Concerning the diagnosis profile for cluster C4, the codes with the highest values were linked to pregnancy (ICD9-CM ‘648’, ‘650’, ‘664’, ‘V27’) and hypertension (ICD9-CM ‘401’). The drug profile of C4 showed the consumption of drugs linked to pregnancy ‘B03AA’ (Iron bivalent), ‘H03CA’ (Iodine therapy), and analgesics (‘N02BB’ and ‘N02BE’). Unlike the drug profile associated with the hypertensive population (see profile in Fig. 1 (d)), ATC ‘C09AA’ did not appear as a frequent drug. This might be motivated because certain types of anti-hypertensive drugs are not recommended in pregnancy. For instance, ACE inhibitors and angiotensin receptor blockers (ARBs) are contraindicated since adverse fetal effects have been reported [62]. Table 1 showed that C4 was composed of 15% of CRG-1000, 5% of CRG-5424, and 80% individuals belong- ing to CRG-5192. Note that the presence of hypertensive patients is notable in this cluster. Though healthy and diabetic patients in C4 have the ICD9-CM codes ‘250’ and ‘648’ (Abnormal glucose tolerance of mother complicating pregnancy childbirth or best known as Gestational Diabetes (GD)), no antihyperglycemic drugs were found. Hence, we can characterize patients of C4 as women suffering from complications (mainly hypertension and GD) during pregnancy, but without consumption of antihypertensive and antihyperglycemic drugs.

For complementing the cluster characterization, we also carried out a correlation analysis using the Pearson Correlation Coefficient (PCC). PCC is a numerical value with 0 meaning no linear relationship and 1 indicating high correlation. Thus, we quantify the relationship between the diagnosis/drug profile of each cluster and the corresponding one for each CRG. The resulting PCC values are shown in Table 2. Cluster C1 presented a high relationship with CRG-5192 in terms of both diagnosis and drug profiles, showing PCC values close to 1. Likewise, the profiles of cluster C2 and CRG-1000 presented a high correlation with PCC values > 0.9. The clusters C3 and C5 present a high correlation with the diagnosis profile of CRG-5424 (PCC values > 0.9). However, the drug profile of C5 showed a moderate relationship with the one associated with CRG-5424 (PCC value ≈ 0.6). The reason is that the most common drugs in cluster C5 are insulins (see purple line in Fig. 4 (c) and last column in Fig. 5), which do not correspond with the highest values in the drug profile of CRG-5424 (see Fig. 1 (f)). Finally, the profiles of C4 did not present a high correlation with any profile of CRGs (PCC < 0.55). As argued, C4 was a cluster encompassing patients with clinical characteristics not exclusively assigned to a particular CRG.

**Table 2:** PCCs between the profiles of each cluster (from C1 to C5) and those associated with CRGs (second-fourth columns) considering the test subset. For each cell, the first and second value refer to the PCC for diagnosis and drug profiles, respectively

	Cluster ID	CRG-1000	CRG-5192	CRG-5424
	C1	0.34; 0.59	0.98; 0.98	0.30; 0.61
	C2	0.92; 0.97	0.58; 0.74	0.52; 0.54
	C3	0.27; 0.39	0.31; 0.56	0.98; 0.94
	C4	0.28; 0.40	0.55; 0.43	0.11; 0.25
	C5	0.26; 0.28	0.13; 0.29	0.96; 0.62

Previous findings are corroborated by the age distribution of each cluster. Thus, Fig. 4 (b) showed that clusters C2, C4, and C5 identified younger patients. By contrast, clusters C1 and C3, with profiles more similar to CRG-5192 and CRG-5424, encompassed the older patients. Although the age variable was not used to get the LRs, note that the combination of clinical codes lead to identifying age patterns in the clusters. Another insight to remark is the notable difference in the age distribution associated with clusters of diabetics. Generally, type-I diabetes has been considered a disease affecting children and adolescents, whilst type-II diabetes is related to adults [63].

### Clinical characterization of clusters associated with healthy, chronic and multimorbid patients

**Fig. 6:**
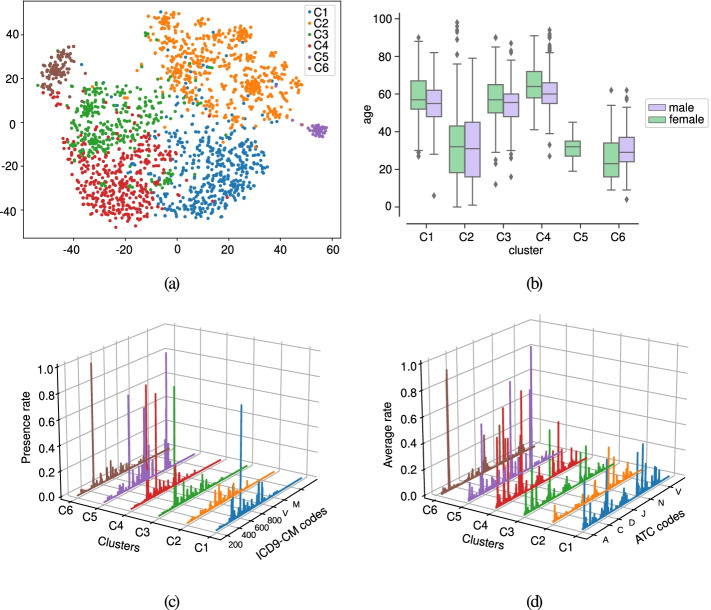
Analysis of clusters considering CRGs-1000-5192-5424-6144 and the test subset. (**a**) Projection of patients by combining DAE, AHC, and t-SNE; (**b**) boxplot of the age for each cluster; profiles considering (**c**) diagnosis codes (**d**) drug codes. Note that each color corresponding to a specific cluster

**Fig. 7:**
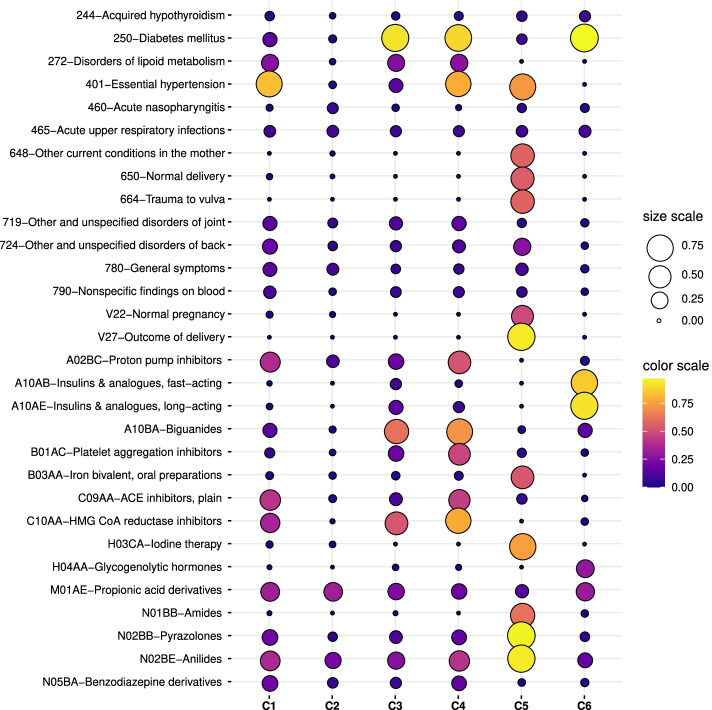
Description of the most frequent clinical codes for the clusters found when considering CRGs-1000-5192-5424-6144 and using the test subset. Each code is represented by a dot whose area and color are proportional to the presence rate

The characterization of the clusters for the second case-study (when considering CRGs-1000-5192-5424-6144) is provided in this subsection. Figure 6 (a) shows the scatter plot of patients when combining DAE, AHC, and t-SNE, identifying clusters as C1 (blue), C2 (orange), C3 (green), C4 (red), C5 (purple), and C6 (brown). As in the previous case, we characterized the clusters based on the age distribution (see Fig. 6 (b)), showing that individuals belonging to clusters C1, C3, and C4 presented higher ages compared to those patients of other clusters. The associated profiles for each cluster considering diagnosis and drug codes are shown in Fig. 6 (c-d), respectively. The description of the clinical codes with the highest presence rate is detailed in Fig. 7.

Considering cluster C1, the analysis of its diagnosis profile showed that the most prevalent diagnosis codes were ICD9-CM ‘401’ and ‘272’. The first one is linked to hypertension and the second one to lipid disorders. Concerning the drug profile, we observed that ATC codes with the highest rates were ‘C09AA’ (a drug for hypertension), ‘C10AA’ (a drug to prevent cardiovascular diseases), and ‘M01AE’ (analgesics). The antihypertensive drug 'C09AA' is one type of selective calcium channel blocker used for preventing the risks associated with blood pressure elevation [59]. Table 3 indicates that the majority of patients in this cluster are associated with CRG-5192. The analysis of profiles showed that patients linked to other CRGs share the drug consumption patterns associated with hypertensive patients (see Fig. 1 (d)). Thus, patients belonging to C1 were characterized as hypertensive.

**Table 3:** Cluster ID, number and percentage of patients associated with CRG-1000, CRG-5192, CRG-5424 and CRG-6144 for each cluster when considering the test subset

Cluster ID	# patients	CRG-1000	CRG-5192	CRG-5424	CRG-6144
C1	514	1	77	5	17
C2	678	81	15	4	0
C3	454	0	1	74	25
C4	376	0	7	10	83
C5	45	18	62	16	4
C6	99	0	0	100	0

By analyzing cluster C2, its diagnosis profile pointed out that no code had a prevalent high presence rate (see Fig. 6 (c)). Most of them presented values below 0.1, with the ICD9-CM codes ‘526’, ‘465’ and ‘780’ reaching the highest presence rate. Regarding the drug profile of cluster C2, most of the ATC codes were related to analgesics (‘M01AE’, ‘N02BE’). Table 3 showed that 81% of patients of C2 belonged to the CRG-1000, while the rest were labeled as CRG-5192 and CRG-5424, with 15% and 4%, respectively. Although there were patients belonging to CRG-5192 and CRG-5424, the drug profile indicated that these patients were not taking drugs for treating hypertension or diabetes. Thus, we can characterize individuals of cluster C2 as healthy patients.

As in the previous case-study, here the diabetic population was also divided into two clusters, C3 (green points in Fig. 6 (a)) and C6 (brown points). Their diagnosis profiles showed ‘250’ as the most frequent ICD9-CM code. Main differences appeared in the drug profiles (see green and brown lines in Fig. 6 (d) and Fig. 7). Cluster C3 was characterized by the consumption of ATC ‘A10BA’ (biguanides) and ‘C10AA’ (statins). As stated, biguanides are key in the treatment of patients suffering from type-II diabetes, while statins are used for lowering the cholesterol level. On the contrary, patients of cluster C6 mostly took two types of insulin with ATC codes ‘A10AB’ and ‘A10AE’. Furthermore, in cluster C6 we identified a moderate presence rate of the ATC code ‘H04AA’, which is a glucose-lowering drug mainly indicated to treat severe hypoglycemia reactions in diabetics treated with insulin [64, 65]. Evidence suggests that between 30-40% of patients with type-I diabetes suffer from one to three episodes of hypoglycemia (insulin excess) per year [66]. Table 3 showed that cluster C3 was mainly composed of patients from CRG-5424 (74%) and the remaining patients were labeled as CRG-6144. Thus, we characterize patients of cluster C6 as diabetics mostly consuming insulin, whilst patients of C3 as diabetics consuming biguanides.

Cluster C4 included patients suffering from multimorbidity, with co-occurring diabetes and hypertension (see profiles in Fig. 6 (c-d) and Fig. 7). The analysis of the diagnosis profile showed ICD9-CM ‘250’ (diabetes) and ‘401’ (essential hypertension) as the most frequent codes in this cluster, both with similar proportions. The evidence suggests that hypertension affects approximately 70% of patients with diabetes [67]. This co-existence of both chronic conditions substantially increases the risk of cerebrovascular and coronary artery diseases [67]. The drug profile showed that the ATC codes with the highest values were ‘C10AA’, ‘C09AA’, ‘A10BA’, ‘B01AC’ and ‘A02BC’. As analyzed in the previous case-study, ‘C09AA’ and ‘A10BA’ are drugs used for treating hypertension and type-II diabetes, respectively. Note that the ATC code ‘B01AC’ (platelet aggregation inhibitors), frequently prescribed to people over 65 for preventing cardiovascular complications [68], was identified in this group with a prevalent presence rate. The co-occurring of several chronic conditions lead to complex profiles, hampering the characterization of the patient’s health status. As presented in Table 3, the majority of patients of C4 were categorized as belonging to CRG-6144.

Patients assigned to cluster C5 had the following most frequent ICD9-CM codes: ‘V27’ (associated with pregnancy), ‘401’ (hypertension), and ‘648’ (linked to complications during pregnancy). Note that the ICD9-CM code ‘648’ includes diabetes mellitus, thyroid dysfunction and abnormal glucose tolerance complicating pregnancy. Regarding the drug profile of cluster C5, the main ATC codes were linked to pregnancy (‘B03AA’) and analgesics (‘N02BB’, ‘N02BE’). Table 3 showed that C5 encompassed patients from the CRG-5192 (62%), 16% of CRG-5424, 18% of CRG-1000 and 4% of CRG-6144. It is interesting to remark that all patients in C5 assigned to CRG-5424 and CRG-6144 were diagnosed with the ICD9-CM codes ‘V27’ and ‘648’. According to the literature [69], between 2-9% of pregnancies are complicated with gestational diabetes, and oral glucose-lowering drugs are not recommended during pregnancy. This is also evidenced through the analysis of the drug profile of C5.

As in the first case-study, we conducted a correlation analysis between the profiles associated with clusters and CRGs. The resulting PCC values are presented in Table 4. It can be noted that profiles of cluster C1 and CRG-5192 resulted quite similar, with PCC values of 0.97. The profiles of cluster C2 were closely related to profiles of CRG-1000 (PCC values ≥ 0.97). Cluster C4, which was mainly composed of patients with multimorbidity, presented the highest correlation with profiles of CRG-6144 (PCC value ≥ 0.98). Cluster C5, which encompasses pregnant women, presented low PCC values with all profiles associated with CRGs. It seems reasonable, since pregnant women are neither patients with chronic conditions nor healthy patients in the sense that there is no prevalent clinical condition.

**Table 4:** PCCs between the profiles of each cluster (from C1 to C6) and those associated with CRGs (second-fourth columns) considering the test subset. For each cell, the first and second value refer to the PCC for diagnosis and drug profiles, respectively

Cluster ID	CRG-1000	CRG-5192	CRG-5424	CRG-6144
C1	0.37; 0.62	0.97; 0.97	0.42; 0.69	0.80; 0.87
C2	0.98; 0.97	0.42; 0.72	0.35; 0.53	0.35; 0.49
C3	0.24; 0.39	0.32; 0.61	0.99; 0.96	0.88; 0.93
C4	0.23; 0.32	0.70; 0.75	0.82; 0.84	0.99; 0.98
C5	0.24; 0.45	0.49; 0.48	0.13; 0.28	0.30; 0.28
C6	0.27; 0.30	0.10; 0.19	0.94; 0.55	0.73; 0.24

Regarding diagnosis profiles of clusters C3 and C6, they presented high correlation with that of CRG-5424 (PCC values ≥ 0.94). On the contrary, the drug profile of C6 showed a moderate correlation with the one associated with CRG-5424 (PCC value of 0.55). Taking into account that all patients of C6 were assigned to CRG-5424 (see Table 3) and the PCC in the drug profile is moderate, it is reasonable to ascertain the clinical evidence behind this fact. According to the literature there are two main types of diabetes, type-I and type-II. Broadly speaking, individuals with type-I may not produce insulin, whereas patients with type-II do not produce enough insulin or the produced insulin does not work properly. This particularity also explains differences in the drug treatments. Type-I is managed by taking insulin to control blood sugar, while biguanides are the first-line treatment for type-II. A deeper analysis of ICD9-CM codes showed that all patients of C6 were diagnosed with type-I diabetes. We must highlight that CRG-5424 gathers individuals diagnosed with diabetes type-I and type-II. Therefore, in the drug profile associated with CRG-5424, the most frequent ATC codes are ‘A10BA’ (biguanides for treating diabetes type-II), ‘C10AA’ (drug for preventing cardiovascular diseases) and ‘A10AE’ (insulin for treating diabetes type-I). However, taking into account that the majority of diabetics in CRG-5424 are of type-II, the drug profile is more representative of the drugs used for treating type-II. Since our approach was able to find patients of type-I (cluster C6) and type-II (cluster C3), the correlation analysis between the drug profile of each cluster and the corresponding one of CRG-5424 provided a high PCC value for cluster C3 while it was moderate for C6.

The clinical characterization showed that clusters C1, C3, and C4 gathered patients suffering from chronic conditions (hypertension, diabetes, and multimorbidity), whereas the rest of the clusters were composed of healthy patients (C2), pregnant women (C5), and insulin-dependent diabetics (C6). Age distribution patterns validated that chronic patients tend to be older people, showing a link between age and chronicity. Evidence also suggests that chronic diseases are more prevalent in older populations, which is in line with the presented results [5].

### Synthetic patients for visual validation of chronic patterns

In this subsection, we describe a proof-of-concept with synthetic patients for the visual characterization of the patient’s health status through LRs. Toward that end, we combine the LRs and t-SNE for projecting real-world patients onto two-dimensional space. Several methods have been used to model patients’ health status evolution in the literature through the use of longitudinal EHR-based data [70, 71]. We did not follow these approaches since multiple records of the same patient over time are not available in our dataset. We propose a first approximation of tracking patients’ health status based on the mapping of synthetic patients. To build the synthetic patients, we firstly identified the most representative clinical codes associated with each cluster. In our case, we selected the first five codes with the highest presence rate values for diagnosis and drug profiles (see Figs. 5 and 7 for the first and second case-study, respectively). We construct simple synthetic patients and complex synthetic patients for each cluster. The goal was twofold: *(i)* to check which ICD9-CM/ATC codes are associated with a specific clinical condition, and *(ii)* to map the health status trajectory of each patient on a two-dimensional plot. This allows us to provide the clinicians with a visual tool to identify health status evolution by incorporating clinical codes.

**Fig. 8:**
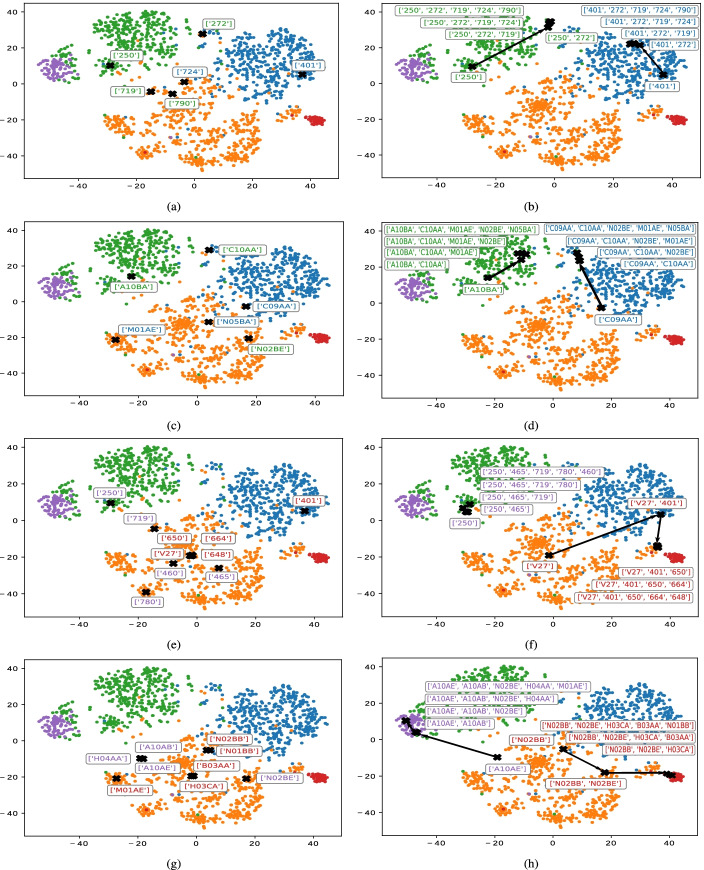
Visualization of simple synthetic patients (left panels) and complex synthetic patients (right panels) when considering CRGs-1000-5192-5424 and: (**a**-**b**) diagnosis codes of C1 and C3; (**c**-**d**) drug codes of C1 and C3; (**e**-**f**) diagnosis codes of C4 and C5; (**g**-**h**) drug codes of C4 and C5. Each color corresponds to a specific cluster: C1 (blue), C2 (orange), C3 (green), C4 (red), C5 (purple)

A simple synthetic patient $\mathbf {p}_{s} \in \mathbb {R}^{D}$ is represented as a vector of length *D* with zeros values, setting just ‘1’ in the element corresponding to a certain target code. This target code corresponds to one of the ICD9-CM/ATC codes with the highest average rate in the profile of each cluster. Thus, each simple synthetic patient **p**_*s*_ is characterized by just one code. Taking as an example the drug profile of C3 when considering CRGs-1000-5192-5424, the code with the highest presence rate is ATC ‘A10BA’. Hence, the first simple synthetic patient is defined by a vector $\mathbf {p}_{s}^{(1)}$ with zero values and only one ‘1’ in the element linked to the ATC ‘A10BA’. The second simple patient $\mathbf {p}_{s}^{(2)}$ has a value of ‘1’ in the element corresponding to the ATC code ‘C10AA’. The rest of the simple synthetic patients are built in the same way. We show the simple synthetic patients (identified by the ’x’ marker) associated with clusters {C1 and C3 } and {C4 and C5 } in the corresponding rows of the left panels of Fig. 8. For simplicity, and to highlight new insights extracted from this study, synthetic patients linked to cluster C2 (who were characterized as healthy patients) were not shown. Since there are common simple synthetic patients between clusters, we just visualize one of them on the plots. For instance, since the ICD9-CM ‘272’ is one of the most frequent codes in the diagnosis profiles of clusters C1 and C3, this code is depicted once.

In order to perform a faithful picture of real-world chronic patients, a complex synthetic patient $\mathbf {p}_{c} \in \mathbb {R}^{D}$ is progressively constructed. Towards that end, we sequentially take into consideration clinical codes ordered according to the highest presence rate in a particular cluster. Formally, our representation of the *i*-th complex synthetic patient is built by aggregating *N*_*i*_ vectors associated with different simple synthetic patients, *i.e.,*$\phantom {\dot {i}\!}\mathbf {p}_{c}^{(i)} = \sum _{j=1}^{N_{i}} \mathbf {p}_{s}^{(i,j)}$, with *N*_*i*_≥2. Note that *N*_*i*_ corresponds to the number of different target codes selected, which increases during the procedure of creation of complex synthetic patients. As an illustrative example, we take the most frequent drug codes in cluster C3 (see Fig. 7). The first complex synthetic patient $\mathbf {p}_{c}^{(1)}$ for cluster C3 when considering just drug codes is represented as a vector with all zero values excepting ‘1’ in the elements linked to ATC ‘A10BA’ and ‘C10AA’. The second complex synthetic patient $\mathbf {p}_{c}^{(2)}$ for C3 is represented as a vector with all zero values excepting those in the elements linked to ATC ‘A10BA’, ‘C10AA’ and ‘M01AE’. Once the complex synthetic patients are constructed, they are displayed on the right panels of Fig. 8.

In Fig. 8 (a-d), we displayed the projection of synthetic patients associated with clusters C1 (blue) and C3 (green), characterized as hypertensives and diabetics, respectively. Note that synthetic patients associated with ICD9-CM codes are projected on Fig. 8 (a-b), and ATC codes in Fig. 8 (c-d). A visual inspection of Fig. 8 (a) showed that simple synthetic patients corresponding with ICD9-CM ‘250’ and ‘401’ were placed on the clusters of diabetics (green points) and hypertensives (blue points), respectively, whereas the rest were located in the cluster of healthy patients (orange points). This pointed out the codes most related to diabetes and hypertension (ICD9-CM ‘250’, ‘401’) and those codes with limited importance (ICD9-CM ‘719’, ‘724’, ‘790’) when they are considered individually for characterizing chronic populations. The analysis of complex patients showed how the aggregation of diagnosis codes provided a visual progression of the mapping associated with complex synthetic patients on the two-dimensional space, thus displaying a potential patient health status evolution. Specifically, in Fig. 8 (b), we showed the complex synthetic patients (depicted with a black arrow) for the diabetic and hypertensive clusters. The code ICD9-CM ‘250’ set the starting point for the synthetic patient in the cluster of diabetics (C3). The aggregation of new diagnosis codes allows us to display a visual progression by approaching the complex synthetic patient to the cluster of hypertensives as *N*_*i*_ increases. The complex patients starting in the hypertensive cluster with the ICD9-CM ‘401’ code showed a similar trend but approaching to the diabetic’s cluster.

Following a similar approach, we use synthetic patients associated with ATC codes in Fig. 8 (c-d). Note how the synthetic patients linked to ATC ‘A10BA’ were mapped on the region associated with diabetic patients (green points), while patients with ATC ‘C09AA’ were on the hypertensives region. Furthermore, it was notable that ATC ‘C10AA’ (see Fig. 8 (c)) was located at the upper part of the scatter plot, far from healthy patients and between diabetics and hypertensives. As shown in the profiles, ‘C10AA’ is a frequent code in clusters that gather chronic patients and it is commonly recommended for preventing cardiovascular diseases [60]. The rest of the synthetic patients (with ATC codes ‘M01AE’, ‘N02BE’, ‘N05BA‘) were mapped on different regions in the cluster of healthy patients. Regarding the synthetic complex patients, two visual progressions associated with clusters C1 (hypertensives) and C3 (diabetics) were identified (see Fig. 8 (d)). The first route started in the position linked to ATC ‘A10BA’ and moved to the upper part of the scatter plot when the second target code (‘C10AA’) was included (see the arrow pointing upwards). The mapping of the route of the second synthetic complex patient started in the position of ATC ‘C09AA’ and reached out to the blue points in upper positions with the aggregation of the target code ATC ‘C10AA’. It is important to remark that even with the addition of new target codes to the synthetic complex patient, the visual progression is almost unaltered. This supports the insight about the low relevance of the last four codes for characterizing these chronic conditions.

By continuing the analysis, we depicted the synthetic patients associated with the diagnosis and drug codes for clusters C4 (red points) and C5 (purple points) in Fig. 8 (e-f) and (g-h). As stated, C4 was composed of women with complications during pregnancy (including hypertension and gestational diabetes) and C5 gathered diabetics who mainly consumed insulin. In Fig. 8 (e), we observed that the simple synthetic patients linked to C4 are characterized by ICD9-CM codes ‘401’, ‘V27’, ‘650’, ‘664’ and ‘648’. As expected, the mapping of the simple synthetic patient with the target code ‘401’ was placed on the cluster of hypertensives (blue points), whereas the remaining codes linked to pregnancy were placed on the region linked to patients characterized as healthy. For cluster C5, the synthetic simple patient with the target code ‘250’ was projected on the cluster of diabetics, whereas the rest of the simple synthetic patients were also mapped in the cluster of healthy patients. In Fig. 8 (f), we observed the visual progression of the complex synthetic patients linked to C4 and C5. The first one started in the position of ICD9-CM ‘V27’ (mapped within the healthy cluster), but with the inclusion of the second target code (ICD9-CM ‘401’), the complex synthetic patient is mapped on the cluster of hypertensives. The aggregation of more target codes (‘650’, ‘664’, ‘648’) moved the synthetic patient mapping to an area close to cluster C4. Note that only using diagnosis codes, the complex synthetic patients were not located within C4. In the case of C5, we observed in Fig. 8 (f) that all complex synthetic patients were mapped on the area associated with diabetics (green points).

A visual inspection of Fig. 8 (g) showed that simple synthetic patients linked to C4 and C5 drug codes were mapped on the cluster of healthy patients (orange points). This was interesting since there were drugs related to insulins (ATC ‘A10AB’, ‘A10AE’) which are directly linked to the diabetes type-I treatment. However, the analysis of complex synthetic patients associated with clusters C4 and C5 provides an interesting progression (see Fig. 8 (h)). The first complex synthetic patient was mapped on the position of the most frequent ATC target code in C4 (‘N02BB’) and moved to the position where the target code ‘N02BE’ was projected, within the area of healthy patients. But the effect of aggregating ‘H03CA’ and ‘B03AA’ (drugs used during pregnancy) was remarkable, moving towards cluster C4 (red points). This highlighted the importance of certain drug codes in the characterization of cluster C4. The case of the complex synthetic patient linked to C5 showed that the aggregation of the ATC codes ‘A10AE’ and ‘A10AB’ produced a visual progression from the area linked to the healthy cluster to the one of the diabetic’s cluster. Remark that the aggregation of more drug target codes (‘H04AA’, ‘M01AE’, ‘N02BE’) did not show great influence in the previous visual progressions.

## Discussion

In this paper, we analyzed the potential of using DAEs aiming to create LRs for characterizing chronic patients based on binary data associated with diagnosis and drug codes. Our goal is not only to reduce dimensionality but also to maintain meaningful patterns of data and obtain effective representations to be used in subsequent clinical tasks such as clustering and visualization.

In the first part of this work, the LRs were used to find clusters of patients with similar clinical characteristics, allowing us to distinguish patients linked to different health statuses. Towards that end, *k*-means and the AHC using Ward linkage were considered, with AHC presenting the best clustering performance according to the analysis of CVI. Next, we combined DAEs and t-SNE to show the potential of our approach to representing high-dimensional data into a two-dimensional space. Mapping on two dimensions resulted to be effective for displaying the health status of patients with chronic conditions, as well as for visualizing the progression of their health status as new clinical codes are considered.

Two case studies were analyzed in this paper, the first one considered healthy, hypertensive and diabetic patients (CRGs-1000-5192-5424) and the second one included multimorbid patients (CRG-1000-5192-5424-6144). As previously stated, the inclusion of the CRG-6144 increased the clinical complexity of the second case-study compared to the first one. This has an impact on the clustering performance (obtaining lower CVI values in the second-case study) and on the visualization performed in the two-dimensional space (more overlapping between clusters). Referring to the clinical findings, we highlight that our approach led to identify clusters of patients with particular clinical conditions which, according to the CRG, were part of a more general health status. In particular, we identify two new subgroups of interest: *(i)* a subgroup of diabetics characterized by the consumption of insulin, directly linked to type-I diabetes (by remarking that none ICD9-CM code for this specific kind of diabetes was used during training); and *(ii)* a cluster of pregnant women who presented complications during pregnancy, including hypertension and gestational diabetes.

In the second part of this work, a proof-of-concept based on synthetic patients was proposed for visualizing patient’s health status progression. The construction of the complex patients and its mapping on a two-dimensional space allowed us to create a first approximation of the real-world patient’s health status evolution. With the aggregation of more clinical codes to the synthetic patients, we could observe the impact of certain codes to lead a clinical condition *A* to another *B* (drawing a route). This could improve the clinical decision-making process, allowing the prediction of a worsening in the health status according to the trend in the path mapped as new drugs and diagnoses are considered. Unsupervised methods as those considered in this study show great potential to discover individuals with a higher risk of developing diabetes or hypertension using clinical data. Data-driven methods shift towards personalized healthcare, allowing to reduce healthcare costs and through the identification of patients with high consumption of resources, such as those affected by chronic conditions. Also, the current approach can be extrapolated to other scenarios (with other clinical conditions), becoming key to finding groups of patients with complex health conditions and studying the influence of different clinical characteristics in the patient’s health status evolution.

Finally, it is relevant to point out that we only used data collected during a natural year. Experiments using data associated with different years are out of the scope of the paper, and they have been considered as future work. With the availability of longitudinal data of several years, we could perform a deeper analysis of the health status progression aiming to allow practitioners to visually examine the mapping of a real-world patient and statistically determine which is the most appropriate treatment for a disease. To highlight that, in the current work, the considered CRGs were selected because they have assigned a representative number of patients for performing properly the ML training. In future work, with the clinical validation of data by experts and the Ethical Committee permission of the UHF, we could consider other CRGs that guarantee a representative number of patients. With the new advances in data synthetic generation in other domains, we could explore more sophisticated ML methods in conjunction with the proposed in the current work. We also plan to work with temporal data for several years, to tackle the progression of the health statuses. This would also allow us to identify time-related disease associations after grouping similar trajectories and study the main events associated with the particular health status evolution of each patient.

## Conclusions

This paper leveraged the potential of using DAEs to create the LRs associated with high-dimensional patient data. On the one hand, experimental results showed that LRs are beneficial to obtain clusters of patients with similar clinical characteristics. On the other hand, the combination of DAEs with mapping methods proved to be an effective visual tool for characterizing chronic patients. In particular, the proof-of-concept based on synthetic patients provided a first approximation of the visualization of the health status progression using ICD9-CM and ATC codes.

Our approach allows practitioners to examine the two-dimensional space and the projection of their patients, aiming to identify disease patterns and monitor the patient’s health status evolution, even proposing early interventions for controlling the disease evolution. Hence, it is possible to determine the factors associated with the onset and progression of chronic conditions. It is important to remark that early interventions can help the physicians to give more specialized treatments, improve the patient’s satisfaction and plan health resources, significantly reducing the economic burden associated with chronic diseases. In conclusion, our approach could support the use of ML models by physicians in daily practice, bringing some light to the decision-making process and the extraction of clinical knowledge. This work opens up interesting lines of research since the addition of new data could lead to a better characterization of the dynamic course of the health status.

## Supplementary Information

Additional file 1. Additional file for learning and visualizing chronic latent representations using electronic health records.

## Data Availability

The data used for this research comprises confidential patient health information. The data obtained from the University Hospital of Fuenlabrada are protected and cannot be released publicly.

## Abbreviations

AE: Autoencoder
AHC: Agglomerative Hierarchical Clustering
ANN: Artificial Neural Network
ATC: Anatomical Therapeutic Chemical Classification System
CRG: Clinical Risk Group
CVI: Cluster Validity Index
DAE: Denoising Autoencoder
EHR: Electronic Health Record
ICD9-CM: International Classification of Diseases Ninth Revision-Clinical Modification
LR: Latent Representation
ML: Machine Learning
PCC: Pearson Correlation Coefficient
SPN: Salt and Pepper Noise
t-SNE: t-distributed Stochastic Neighbour Embedding
UHF: University Hospital of Fuenlabrada
ZMN: Zero Masking Noise
