# Supplementary material for: Learning and visualizing chronic latent representations using electronic health records
Source: BioData Min. 2022 Sep 5;15:18. doi: 10.1186/s13040-022-00303-z (PMC9446539; doi:10.1186/s13040-022-00303-z)
Supplement: Supplementary file 1 — Additional file 1. Additional file for learning and visualizing chronic latent representations using electronic health records. [file 13040_2022_303_MOESM1_ESM.pdf]

SHORT REPORT ADDITIONAL FILE

Additional file for Learning and Visualizing Chronic Latent Representations using Electronic Health Records

David Chushig-Muzo*, Cristina Soguero-Ruiz, Pablo de Miguel Bohoyo and Inmaculada Mora-Jiménez

*Correspondence: david.chushig@urjc.es
Full list of author information is available at the end of the article

Figures

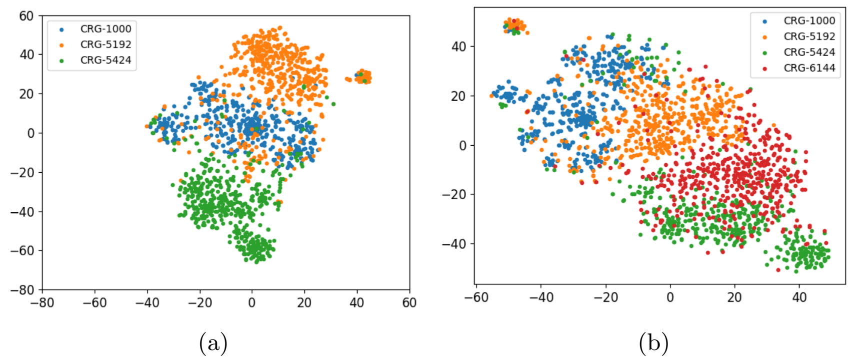

Figure 1: Projection of patients when applying t-SNE on the first 20 components of Principal Component Analysis for: (a) CRG-1000-5192-5424; (b) CRG-1000-5192-5424-6144.

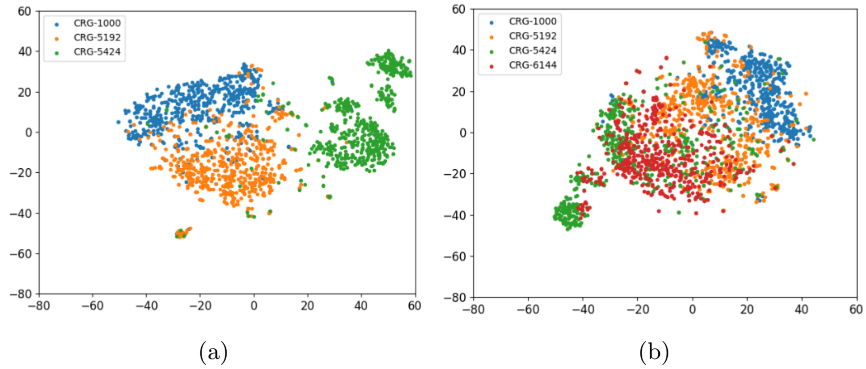

Figure 2: Projection of patients when applying t-SNE on the first 20 components of Factor Analysis for: (a) CRG-1000-5192-5424; (b) CRG-1000-5192-5424-6144.

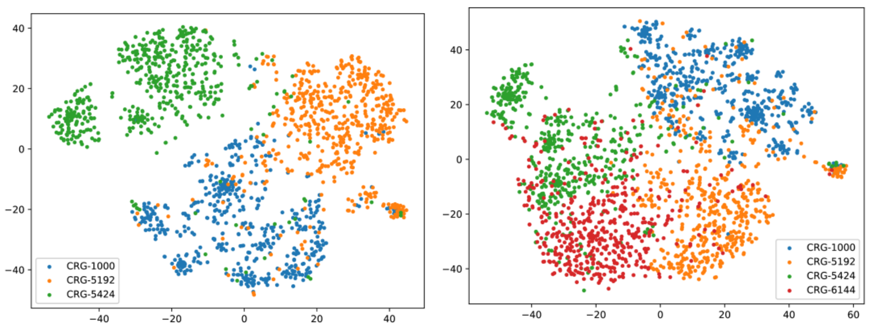

(a) (b)

Figure 3: Projection of patients when applying t-SNE on the 20 latent dimensions of the hidden layer in Denoising Autoencoder when trained using SPN and FN=10 for: (a) CRG-1000-5192-5424; (b) CRG-1000-5192-5424-6144.

We present in this file of supplementary material the mappings obtained when using both linear methods (Principal Component Analysis and Factor Analysis, two of the most used dimensionality reduction methods) and a nonlinear method (Denoising Autoencoder) for reducing the high-dimensionality in the original data to 20 features.

The analysis was conducted in two stages. First, the dimensionality reduction methods are used to transform the high-dimensional space into a low dimensional space. The number of selected components using Principal Component Analysis, and of factors when using Factor Analysis is 20, coinciding with the number of selected latent dimensions. Then, the t-SNE technique is used to project the observations embedded in the transformed features into a two-dimensional space. The projection results are shown in the tree figures in this file. Left panel in each figure refers to the projection of patients assigned to the healthy group (CRG-1000) and to two groups with a single chronic condition (CRG-5192 and CRG-5424). Right panel in each figure illustrates the mapping when considering also patients with two co-occurring chronic conditions (CRG-6144). Note that mappings using DAE improve the visualization when considering patients with more than one chronic dominant condition, evidencing the advantage of nonlinear methods versus linear dimensionality reduction methods.
